# Further studies on the biological activity of hazelnut allergens

**DOI:** 10.1186/s13601-015-0066-7

**Published:** 2015-07-17

**Authors:** F. Blanc, H. Bernard, S. Ah-Leung, L. Przybylski-Nicaise, P. Stahl Skov, A. Purohit, F. de Blay, B. Ballmer-Weber, P. Fritsche, M. Fernandez Rivas, I. Reig, A. Sinaniotis, E. Vassilopoulou, K. Hoffmann-Sommergruber, S. Vieths, N. Rigby, C. Mills, K. Adel-Patient

**Affiliations:** INRA-CEA, Service de Pharmacologie et d’Immunoanalyse, Laboratoire d’Immuno-Allergie Alimentaire, Bât. 136–CEA de Saclay, 91191 Gif-sur-Yvette, France; RefLab ApS, Copenhagen, Denmark; Hôpitaux Universitaires de Strasbourg, Strasbourg, France; University Hospital Zurich, Zurich, Switzerland; Hospital Clinico San Carlos, Madrid, Spain; University of Athens & “Sotiria” Regional Chest Diseases Hospital of Athens, Athens, Greece; University of Nicosia, Nicosia, Cyprus; Medical University of Vienna, Vienna, Austria; Paul-Ehrlich-Institut, Langen, Germany; Institute of Food Research, Norwich, UK; University of Manchester, Manchester, UK

**Keywords:** Food allergy, Hazelnut, Purified allergens, Sensitization patterns, Biological activity, Cor a 1, Cor a 8

## Abstract

**Background:**

Sensitization to hazelnut allergens vary depending on the geographic origin and age of the patients. The objective of this study was to further investigate the allergenic activity of hazelnut allergens using sera from patients recruited in various European regions and presenting different sensitization patterns to hazelnut proteins.

**Methods:**

Natural Cor a 11 and Cor a 9 were purified from hazelnut whereas Cor a 1 and Cor a 8 were produced as recombinant proteins (rCor a 1.04 and rCor a 8). Sera from hazelnut allergic patients were collected in France (*n* = 5), Switzerland (*n* = 2), Greece (*n* = 11) and Spain (*n* = 3), within the Europrevall project. Total and allergen-specific IgE were quantified by enzyme allergosorbent test and IgE immunoblot were performed using pooled sera from birch-pollen endemic region or from Greece. Histamine Release (HR) assays were performed with stripped basophils passively sensitized with individual sera and challenged by a hazelnut extract or the different hazelnut allergens.

**Results:**

As previously described, hazelnut allergic patients from Mediterranean countries are mainly sensitized to the nsLTP Cor a 8 whereas patients from France and Switzerland are sensitized to pollen-related allergens. Interestingly, an intermediate profile was evidenced in patients from Madrid. Hazelnut 7S globulin (Cor a 11) and 11S globulin (Cor a 9) were found to be minor allergens, recognized only by patients from Mediterranean countries. The biologic activity of the 4 tested allergens, analysed by HR assay, further confirmed the sensitization patterns, but also demonstrated the very high elicitation potency of Cor a 8.

**Conclusions:**

This work, extending previously published researches, represents a step towards the better understanding of the complexity of hazelnut allergy and provides new data on the biological activity of hazelnut allergens and extracts.

**Electronic supplementary material:**

The online version of this article (doi:10.1186/s13601-015-0066-7) contains supplementary material, which is available to authorized users.

## Background

Recently, Europrevall data demonstrated that hazelnut (*Corylus avellana*) is one of the most common trigger of IgE mediated food allergies. In fact, a very high prevalence rate of sensitization to hazelnut was evidenced in adults, reaching a mean value of 9.3 % of the general population aged 20–57 years in eight European centers [1]. Prevalence was however variable between centers, ranging from 1.27 % in Reykjavik to 17.79 % in Zurich. Hazelnut allergy was also the most reported food allergy in Europrevall clinic survey (32 %), with frequencies ranging from 68.4 % in Vilnius to 5.7 % in Madrid [2]. Hazelnut allergy involved different kind of allergens, that is type 1 (“complete”) or type 2 (“incomplete”) allergens, leading to different symptoms. Allergic reaction induced by type 2 hazelnut allergens occurred in patients primary sensitized to pollen allergens, mainly from birch. The later consumption of hazelnut, containing proteins homologous to the sensitizing aeroallergen, will lead to symptoms localized to the oral cavity (Oral Allergy Syndrome–OAS; [3]). Type 1 allergens are able both to sensitize and to elicit an allergic reaction and induce more severe reactions such as urticaria, angiodaema or anaphylaxis. Those symptoms are evidenced in “true food allergic” patients [4, 5].

IgE sensitization to hazelnut then combined both IgE recognition of allergens cross reacting with pollen allergens of the PR-10 family, i.e., Bet v 1-related (Cor a 1), and profilin (Cor a 2), and true food allergens such as the non-specific Lipid Transfer Protein (LTP) Cor a 8 and the seed storage proteins Cor a 9 (11S globulin), Cor a 11 (7S globulin) and Cor a 14 (2S albumin). In fact, different proteins have been involved as allergens in hazelnut, the prevalence of specific ones being related to geographical origin and age [4, 6, 7]. Predominance of sensitization to the Bet v 1 related protein Cor a 1.04 is mainly observed in birch-endemic region, i.e., Northern and Central Europe, whereas Cor a 8 is more implicated in non-birch endemic Mediterranean regions such as Spain and Greece [2, 4, 5, 8, 9]. Sensitization to Cor a 8 purified from hazelnut was also evidenced in children from birch-endemic area, i.e., the Netherlands, in which it was associated to more severe allergic reaction upon challenge [9]. However, this was not confirmed in a later study using recombinant Cor a 8 (rCor a 8), suggesting a contamination of the natural Cor a 8 by other seed storage proteins [6, 8, 10].

Hazelnut allergy in birch-endemic region was also demonstrated to exhibit age-related sensitization profiles with different clinical outcomes. Young children (<2 years) demonstrated more severe reaction with systemic reaction, without associated birch-pollen allergy. Sixty five percent of these children were sensitized to Cor a 9. Sensitization to Cor a 9 decreased with age, and OAS predominated in adults, 90 % of which were sensitized to Cor a 1.04 [6]. In the same birch-endemic region, out of 34 children of less than 1-year-old demonstrating atopic dermatitis, 20 demonstrated IgE reactivity to hazelnut and 15 to peanut. Of the hazelnut sensitized children, 75 % demonstrated anti-Cor a 9 IgE, whereas no sensitization to Cor a 1 nor Cor a 8 was evidenced [11]. Sensitization to Cor a 9 and Cor a 11 was further evidenced mainly in children with systemic reaction in the same birch-endemic area [7]. Sensitization to Cor a 9 and/or Cor a 14 was later demonstrated to identify almost 90 % of children with generalized reactions [12]. In a Dutch hazelnut-sensitized population, sensitization and levels of specific IgE to Cor a 9 and to the 2S albumin Cor a 14 were also significantly more common in both children and adults patients demonstrating objective symptoms compared to patients with no or subjective symptoms [10]. Sensitization to the basic subunit of an isoform of Cor a 9 was also recently described in an Italian pediatric population demonstrating severe symptoms (urticarial, angioedema and/or anaphylaxis), and may be involved in cross-reactivity between nuts and legumes. In this population, Cor a 8 and Cor a 9 were also recognized by 47 and 80 % of the patients, respectively [13].

The objective of the present study was then to further assess the allergenic activity of the hazelnut allergens using Histamine Release (HR) assays and sera from patients recruited in various European regions, i.e., France, Switzerland, Spain and Greece. Sensitization patterns to hazelnut proteins were first assessed by specific IgE measurement and IgE immunoblots to confirm regional variations.

## Methods

### Allergic patients

Sera of patients with a self-reported hazelnut allergy have been collected within the Europrevall project in Switzerland (Zurich), East of France (Strasbourg), Greece (Athens) and Spain (Madrid) (Table 1; *n* = 21). A written informed consent was obtained before the serum collection and the performed experiments were approved by the corresponding local ethical committees.

**Table 1:** Data from Hazelnut allergic patients

Source	Reference	Age	Gender	Hazelnut allergy			Other food allergies	Other allergies
				symptoms	SPT	CAP specific IgE (kU/L)		
Strasbourg, France	1	49	f	Rhinitis, Conjunctivitis, Asthma	++	9.7	kiwi, ananas	no
	2	17	m	Rhinitis, Conjunctivitis	++	>100.0	peanut, apple, celery, soya, walnut, sesame, kiwi, carrot, tomato, cherry, fig	cat
	3	25	f	Rhinitis, Conjunctivitis	++	31.2		no
	4	30	m	Rhinitis, Conjunctivitis, Asthma	+	1		house dust mite
	5	33	f	Rhinitis, Conjunctivitis, Asthma	++	>100.0		cat
Athens, Greece	6	42	f		+	1.7	peanut, cabbage, pepper, honey	Resp Allergy Rhinitis
	7	24	f		−	2.6	almond	Resp Allergy Rhinitis, Asthma
	8	21	m	OAS	+	1.0	eggplant	no
	9	36	m	Asymptomatic sensitization	−	3.0	walnut	Resp Allergy Rhinitis
	10	27	m	OAS	++	5.1	peanut, walnut, almond, cashew, pistachio, peach, apple, appricot, mustard, corn	Resp Allergy Rhinitis, Asthma
	11	21	f	OAS	+	0.5	peach, walnut	Resp Allergy Rhinitis
	12	18	f	Asymptomatic sensitization	−	28.5	cabbage, wheat	no
	13			Hazelnut allergy history				
	14	8	m	OAS	++	n/a	egg	no
	15							
	16			hazelnut allergy history				
Madrid, Spain	17	34	f	urticaria + contact urticaria	++	5.1	lentil, chickpea, bean, pea, soybean, sunflower seed, pine nut	grass and olive pollen allergies - rhinoconjunctivitis and asthma
	18	4	m	OAS	++	2.3	walnut	NO
	19	16	f	tongue angioedema	++	7.3	walnut, vetchling	pollen allergy - rhinoconjunctivitis and asthma - due to grass, cypress and plane tree pollens
Zurich, Switzerland	20							
	21							

Asymptomatic sensitization: SPT results (−,+ or ++ according to the SPT/histamine wheal size ratio) or/and specific IgE (CAP) to hazelnut in the absence of clinical symptoms

No information was available for Zurich patients

*OAS* Oral Allergy Syndrom

### Natural allergen purification and recombinant allergens

Natural Cor a 11 and Cor a 9 were purified from hazelnut as described in [14]. Cor a 1.04 and Cor a 8 were produced as recombinant proteins [15] and kindly provided by the EuroPrevall allergen library. Hazelnut proteins correspond to RefLab Hazelnut extract (Histamine release) or were extracted from hazelnut flour (IgE-immunoblot, The Nutt Ranch, Marlborough, New Zealand; Kindly provided by Dr Justin Marsh, University of Manchester) using 0.3 % Sodium Borate, 10 mM EDTA, 0.9 % n-octyl-β-D-Glucoside pH 9.0 buffer. Protein extraction efficiency was about 80 % considering theoretical protein content of hazelnut flour.

### Determination of total and specific IgE concentrations

Total IgE and IgE specific for Cor a 11, Cor a 9, rCor a 1.04 and rCor a 8 were quantified by EAST (enzyme allergo-sorbent test) as previously described [16–18]. Briefly, microtitre plates (96 well microtiter MaxiSorp Nunc, Roskilde, Denmark) were coated by passive adsorption of purified hazelnut proteins (specific IgE) or anti-human IgE antibody (total IgE, clone LE-27) (5 μg/ml in 50 mM phosphate buffer, pH 7.4). After saturation with EIA buffer (0.1 M phosphate buffer, 0.1 % bovine serum albumin, 0.15 M NaCl, 0.01 % sodium azide, pH 7.4), 50 μl per well of serial dilutions of individual serum (1/4 to 1/2500, in EIA buffer) were dispensed. After a 24-h incubation at 4 °C and a wash step, a second anti-human IgE antibody (clone BS17) labelled with acetylcholinesterase (AChE) was used as tracer. After extensive washings, Ellman’s reagent was used as enzyme substrate and the absorbance was measured at 414 nm. Limit of detection, corresponding to the mean background value plus three standard deviation, was 0.1 IU/ml. Total IgE concentration was determined as described in [18] using anti-IgE coated plate and human IgE samples (World Health Organization) at concentrations ranging from 10 to 0.08 IU/ml as standard.

### IgE-immunoblot

SDS-PAGE and IgE immunoblot analyses were performed using apparatus and reagents from Invitrogen (Life Technologies, Carlsbad, CA, USA) following provider’s recommendation. Samples and molecular-weight markers (See Blue prestained marker) were loaded on a NuPage Novex 4-12 % Bis-Tris Gel (1.0 mm). Electrophoresis was performed using XCell SureLock Mini-Cell in MES Buffer with a constant voltage of 200 V during 40 min. After electrophoresis, gels were stained with SimplyBlue™ Safe Stain or proteins were transferred to PVDF membranes for 60 min at 30 V using a XCell II blot module. The membranes were saturated with TBST (20 mM Tris, pH 8.0, 0.25 M NaCl, 0.5 % Tween) supplemented with 5 % milk powder. Immunoblots were performed with a pool of sera specific for Cor a 1.04 from birch-endemic region, or a pool of sera from Athens specific for Cor a 8, Cor a 9 and Cor a 11. Sera diluted 1/40 in TBST supplemented with 5 % milk powder were incubated overnight at 4 °C. Membranes were then washed using TBST, and a human anti-IgE monoclonal Antibody (Star 96P, Serotec) labelled with peroxidase was incubated for 2 h at room temperature. After several washings using TBS (20 mM Tris, pH 8.0, 0.25 M NaCl), the membrane was incubated with the ECL plus Western blotting detection reagent (Amersham GE Healthcare, Buckinghamshire, UK) for 5 min and images then acquired using VersaDoc Imaging System (BioRad, Hercules, CA).

### Basophil histamine release assay

PBMCs were isolated from 20 mL of fresh buffy coats (Blood Bank, National University Hospital of Copenhagen, Denmark) using the lymphoprep isolation method. After isolation PBMCs were washed twice in physiologic saline and IgE were removed by exposing the cell pellet to stripping buffer (RefLab, Copenhagen, Denmark; 4 °C for 5 min). After centrifugation, cells were washed twice with Pipes buffer (RefLab) and finally resuspended to a final volume of 2 mL. Aliquots of 100 μl stripped cells were incubated with 100 μl undiluted patient serum or one sera from non allergic patient as a control for 1 h at 37 °C and then resuspended in 3 mL of a washed erythrocyte obtained from the lymphoprep separation suspension containing IL-3 (2 ng/mL). Twenty-five microliters of the passively sensitized cell suspension was incubated with 25 μl of different concentrations of hazelnut extract or purified hazelnut allergens (Cor a 9, Cor a 11, rCor a 1.04 and rCor a 8) for 1 h at 37 °C on HR-Test Plate (RefLab, Copenhagen, Denmark). Anti-IgE or buffer was used as positive (reference) and negative control, respectively. After washing, released histamine was detected fluorometrically according to the method described by Stahl et al. [19]. A net histamine release > 10 ng/mL was considered as a positive response and histamine release was expressed as percentage of reference release. Results were also given in classes 0–6, taking into account the range of allergen concentration inducing a significant histamine release (the lower this concentration is, the higher the class is).

## Results

### Sensitization pattern

#### Total and specific IgE

**Fig. 1:**
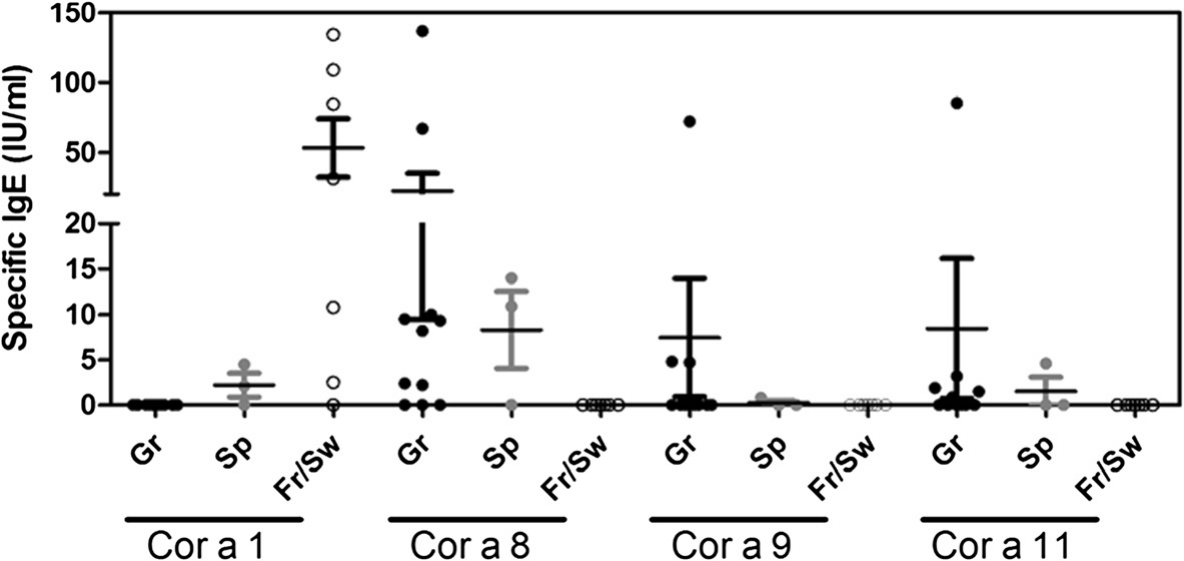
Sensitization pattern depending on the geographical origin of the patient. Specific IgE against Cor a 11, Cor a 9, rCor a 1.04 and rCor a 8 were assayed by EAST

Whatever the regional origin of the patient, total IgE showed a great variability ranging from less than 15 to 4500 IU/mL (Table 2). Patients’ sera were considered positive when specific IgE were detected against at least one of the four allergens, i.e., rCor a 1, rCor a 8, Cor a 9 and Cor a 11. All patient sera from Spain, 9 out of the 11 patient sera from Greece, and 6 out of the 7 patient sera from Switzerland and East of France were positive (Table 2; sensitization pattern depending on geographical area is depicted Fig. 1). Within positive sera, those coming from patients from France and Switzerland demonstrated IgE exclusively directed against rCor a 1.04 whereas none of the positive patient’s sera from Greece demonstrated IgE specific to this allergen. Conversely, most of the positive Greek sera (8 out of 9) had specific IgE directed against rCor a 8. Sera from 2 out of the 3 patients recruited in Spain showed intermediate patterns, with both specific IgE to rCor a 8 and rCor a 1.04. The third one demonstrated only Cor a 9-specific IgE (Patient #18).

**Table 2:** Total IgE and Cor a 11-, Cor a 9-, rCor a 1.04- and rCor a 8-specific IgE in sera, depending on the regional origin of the patients

Origin	Reference	Total IgE (IU/mL)	Specific IgE (IU/mL)			
			Cor a 11 (7S globulin)	Cor a 9 (11S globulin)	rCor a 1.04 (Bet v 1 like)	rCor a 8 (nsLTP)
Strasbourg, France	1	19	–	–	2.5	–
	2	183	–	–	84.7	–
	3	52	–	–	10.8	–
	4	<15	–	–	–	–
	5	757	–	–	134.5	–
Athens, Greece	6	176	–	–	–	10.0
	7	891	1.5	–	–	9.3
	8	<15	–	–	–	2.4
	9	189	1.9	–	–	–
	10	898	–	–	–	8.2
	11	118	–	–	–	–
	12	861	0.9	–	–	137.0
	13	500	3.2	4.7	–	9.5
	14	1027	85.4	72.3	–	2.2
	15	1591	–	4.8	–	67.2
	16	29	–	–	–	–
Madrid, Spain	17	779	–	–	2.1	14.0
	18	72	–	0.8	–	–
	19	4537	4.6	–	4.5	10.9
Zurich, Switzerland	20	275	–	–	31.3	–
	21	4350	–	–	109.2	–

(− = < limit of detection (0.1 IU/mL))

Only few sera had specific IgE directed against either Cor a 11 or Cor a 9. They all came from patients recruited in Greece (5/11 and 3/11 respectively) and Spain (1/3 in each case). The higher levels of specific IgE against these two allergens were demonstrated in a patient from Greece that demonstrated low levels of anti-rCor a 8 IgE (Patient #14).

#### IgE immunoblot

**Fig. 2:**
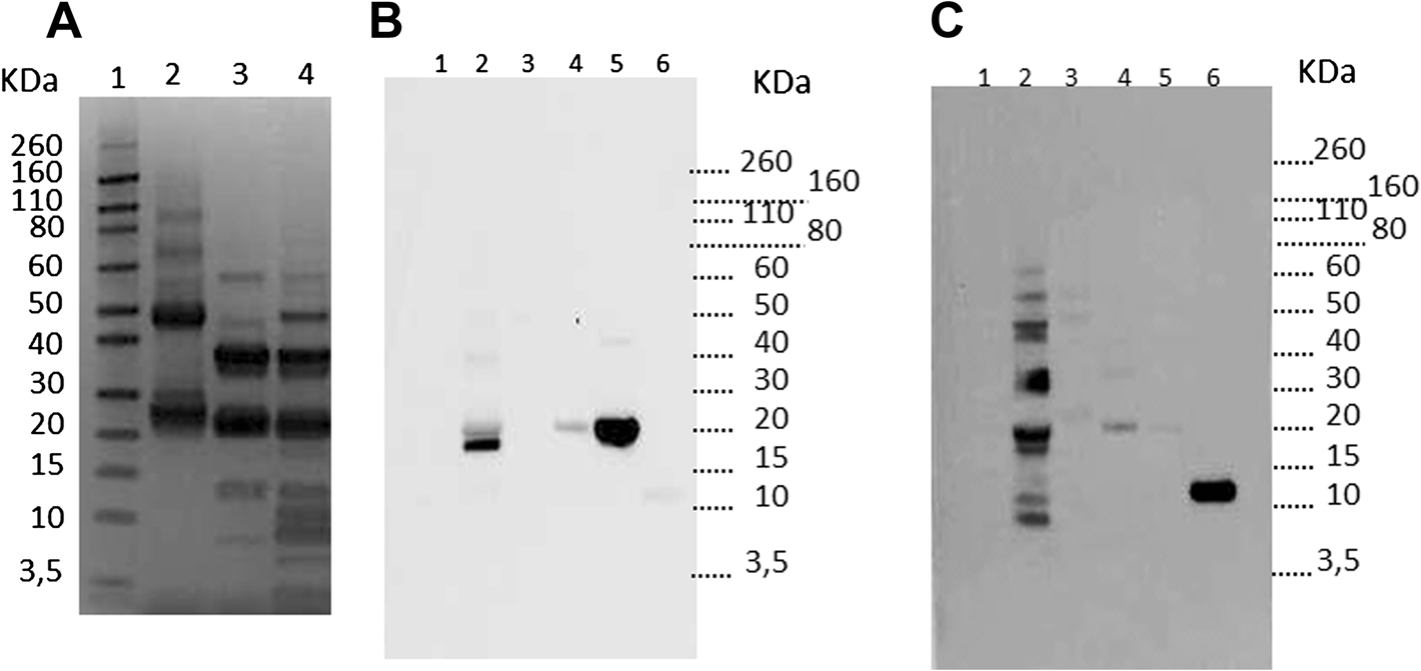
IgE immunoblot using pooled sera from birch endemic region or from Greece. (**a**) Electrophoresis profile of purified Cor a 11 (*lane 2*), purified Cor a 9 (*lane 3*) and hazelnut extract (*lane 4*). IgE immunoblot using pool of sera from birch-endemic region (**b**) or from Greece (**c**) using hazelnut extract (*lane 2*), purified Cor a 11 (*lane 3*), purified Cor a 9 (*lane 4*), rCor a 1.04 (*lane 5*) or rCor a 8 (*lane 6*). Molecular weights (*lane 1*) are indicated

Pools of sera from patients sensitized exclusively to Cor a 1.04 (birch-endemic region pool) or from patients sensitized to Cor a 8, Cor a 9 and Cor a 11 (Greek pool) were constituted to further analyse their sensitization pattern by IgE-immunoblot. Far more bands were revealed using sera from Greece when compared to birch-endemic region pool that demonstrated very restricted recognition pattern. As evidenced using sera from the birch endemic region, Cor a 1 was intensively recognized when analysing purified recombinant allergen (Fig. 2b, lane 5). However, Cor a 1 recognition was far less intense within the complex hazelnut extract (Fig. 2b, lane 2). Conversely, another band, at MW below that of Cor a 1, was more intensively revealed by IgE-immunoblot.

Electrophoresis pattern of purified Cor a 11 and Cor a 9 evidenced several bands (Fig. 2a), as previously described [14]. Cor a 11 polypeptides with apparent MW of 47 KDa were evidenced whereas the 31–35 KDa bands were hardly visible, suggesting degradation of the corresponding entities–certainly as several intense bands between 20 and 30 KDa (Fig. 2a, lane 2). All those Cor a 11-related bands were slightly recognized by IgE from Greek pool sera (Fig. 2c, lane 3), and not recognized at all by IgE from birch-endemic region pool (Fig. 2b, lane 3). Basic (21–25 KDa) and acidic chains (31–35 KDa) of 11S globulin Cor a 9 were also clearly evidenced by electrophoresis (Fig. 2a, lane 3), as the same as lower MW polypeptides between 9 and 12 KDa. Basic and acidic bands were recognised by IgE from the Greek pool, as evidenced both using the purified allergen (Fig. 2c, lane 4) and the hazelnut extract (Fig. 2c, lane 2). IgE immunoblot also demonstrated the intense recognition of rCor a 8 only by the Greek pool sera (lane 6: rCor a 8 and lane 2: hazelnut extract). Another band, at MW below that of Cor a 8, was also revealed by IgE-immunoblot with Greek pool.

### Histamine release

**Fig. 3:**
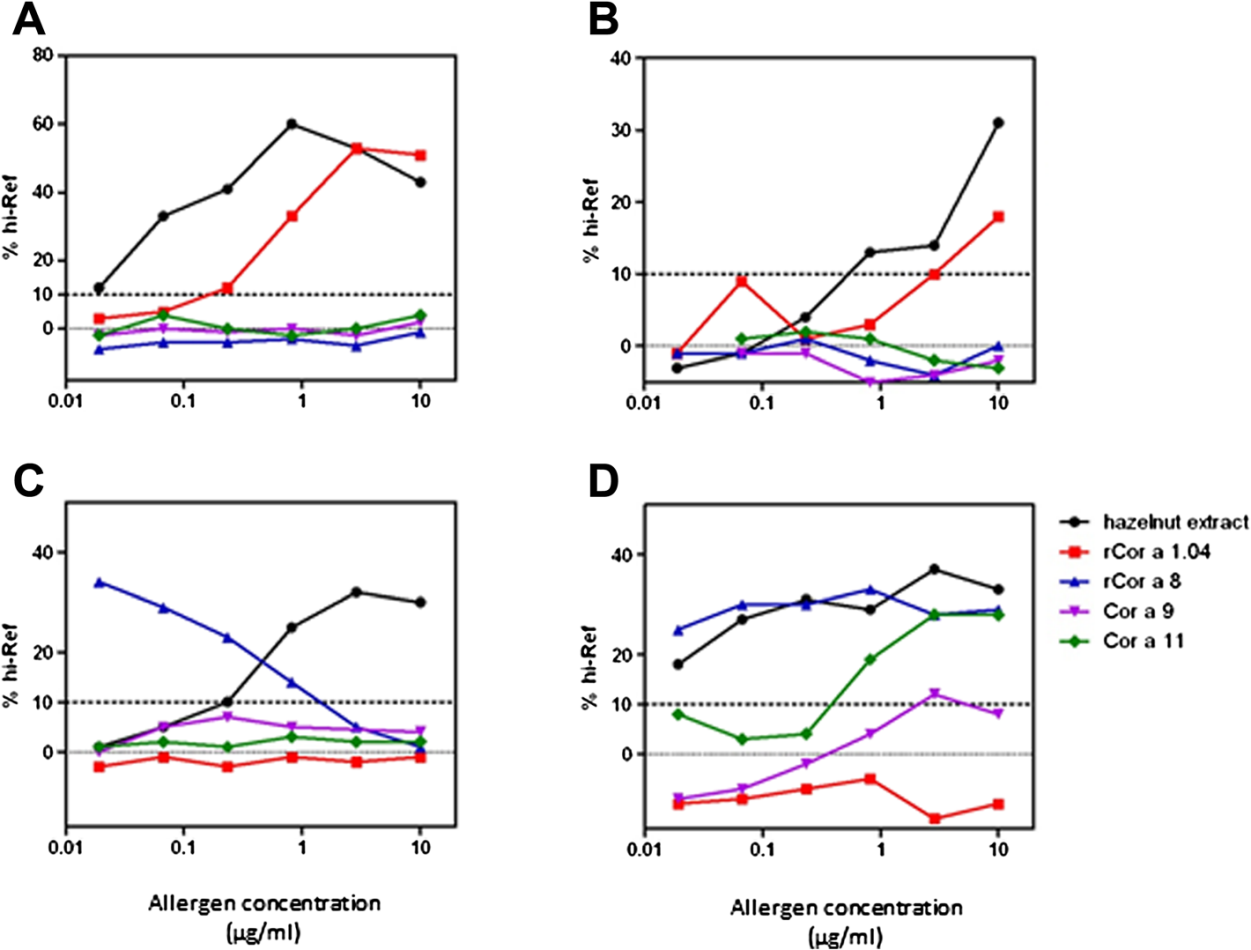
Basophil histamine release induced by hazelnut allergens depending on the geographical origin of the sensitizing sera. Stripped human basophils were passively sensitized with serum #21 (Zurich–**a**), #1 (Strasbourg–**b**), #8 or #12 (Athens, **c** and **d** respectively) and then incubated with increasing concentration of hazelnut allergens, i.e., hazelnut extract (*black*), rCor a 1 (*red*), rCor a 8 (*blue*), Cor a 9 (*purple*) or Cor a 11 (*green*). Released histamine is represented as percentage of reference release, i.e., induced by anti-IgE

**Fig. 4:**
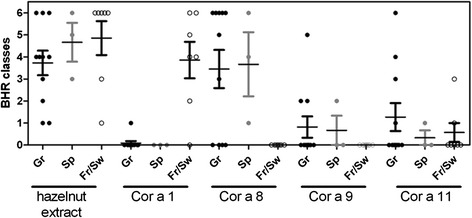
BHR classes depending on the geographical origin of the sensitizing sera and of the purified allergens. BHR class represent the range of allergen concentration inducing a significant histamine release

HR tests were performed for all the sera collected in France/Switzerland (*n* = 7), Spain (*n* = 3) and Greece (*n* = 11). All sera from allergic patients tested induced histamine release after challenge with the hazelnut extract, whereas serum from non allergic patient did not (data not shown). Typical results obtained with 4 sera are shown Fig. 3 and Basophil histamine release classes obtained from all the sera depending of their geographical origin are gathered Fig. 4. Globally, good correlation was observed between IgE titers (Fig. 1) and HR assay. The 2 sera from Greece (#11 and #16) and sera from Strasbourg (#4) negative for specific IgE demonstrated low HR using the hazelnut extract (BHR class 1), whereas no allergen-specific HR was detected for these sera.

Figure 3a and b shows typical results obtained with 1 serum from Switzerland and France, respectively. Among all sera from patients coming from France and Switzerland, 6 out of 7 demonstrated rCor a 1-specific HR, whereas none of them demonstrated rCor a 8, Cor a 9 nor Cor a 11-specific HR, correlating with specific IgE. Sera in Fig. 3 demonstrated high (patient #21, Fig. 3a) or low (patient #1, Fig. 3b) rCor a 1-specific IgE concentrations (Table 2). Additional results using 3 other sera from France with various specific IgE titers are shown in Additional file 1: Figure S1. No clear correlation between rCor a 1-specific IgE concentrations and HR intensity could be evidenced.

On the opposite, sera from Greek patients induced mainly Cor a 8-specific HR, at very low doses of allergen (Fig. 3c and d showing two sera from Greece), suggesting a high elicitation potency of this allergen. All the sera of patients from Spain induced HR after rCor a 8 challenge (even patient #18 who did not demonstrated specific IgE), but not after rCor a 1 challenge, although rCor a 1 specific IgE were detected in 2 of these 3 sera. One of them demonstrated HR after Cor a 9 and Cor a 11 challenge, but at the highest tested doses. Globally, Cor a 9 and Cor a 11-specific HR were less frequent (Cor a 9: 3 sera from Greek patients, 1 serum from Spain; Cor a 11: 4 sera from Greece, 1 serum from Spain, 2 sera from France; Fig. 4). Considering those allergens, HR occurred at higher doses than those observed for rCor a 8 (Fig. 3d).

## Discussion

The results obtained in our adult population confirm that hazelnut allergic patients from Mediterranean countries are mainly sensitized to Cor a 8 whereas patients from Northern Europe are sensitized to pollen-related allergens such as Cor a 1.04 [2, 4, 5, 8]. In fact, in our population, Cor a 1.04 sensitization was clearly evidenced in French and Swiss patients when using EAST or IgE immunoblot with purified recombinant allergen, correlating with the very high frequency of recognition (>80 %) of this allergen within Strasbourg and Zurich cohorts of Europrevall [2]. It is to note however that another IgE-reactive protein is evidenced by IgE immunoblot using Birch-endemic pool, leading to an even more intense signal than Cor a 1 within the hazelnut extract (Fig. 2b). This protein could correspond to another pollen-related allergen such as Cor a 2 (MW 14 KDa vs Cor a 1 MW 17–18 KDa, [4, 20]). In fact, Cor a 2 was also recognized by about 19 % of Zurich and Strasbourg patients within Europrevall cohorts [2], by 26 % of patients form Zurich and Copenhagen [4] and up to 42 % in hazelnut allergic patients from Switzerland or Denmark [8]. Using purified allergens and ImmunoCAP, the IgE concentrations against rCor a 1 were demonstrated to be far higher than that against rCor a 2 [2, 8], but our study suggests that the contents and/or the allergenic activity [21] of the relative allergens within the hazelnut extract have also to be taken into consideration to further assess their relative contribution in hazelnut allergy. This is further confirmed when comparing elicitation potency of rCor a 1.04 vs hazelnut extract (see below).

Conversely, no Cor a 1.04 sensitization was evidenced in the Greek patients whereas Cor a 8 sensitization was observed in 8 out 9 of the sensitized patients from Athens, comforting recent data [2]. Within these 8 patients and considering the allergens we tested, 3 patients were monosensitized to Cor a 8. In a study involving 26 hazelnut patients from Spain, in which 10 patients demonstrated anaphylactic reaction, rCor a 8 was recognized by 62 to 77 % of the patients, most of them being monosensitized, whereas only 1 patient demonstrated IgE against rCor a 1.04 and none against rCor a 2 [5]. Interestingly, the 3 Spanish patients in the present study demonstrated intermediate sensitization profiles, 2 of them demonstrating both rCor a 1.04 and rCor a 8 specific IgE.

We observed sensitization to seed storage proteins Cor a 9 and Cor a 11 in 55 and 33 %, respectively, of the sensitized patients from Athens (*n* = 9), and 1 out 3 patients from Madrid. None of the French nor Swiss patients tested (*n* = 7) demonstrated specific IgE against these proteins. The IgE-immunoblot using the Greek pool confirmed sensitization to Cor a 8, Cor a 9 and Cor a 11. It is to note that purified Cor a 9 used in the present study may also contain isoallergen of Cor a 9, notably its 20.7 KDa IgE reactive subunit recently described in Italian paediatric patients ([13], Fig. 2c). Others bands suggest also sensitization to the 2S albumin Cor a 14 (MW around 9 KDa, Fig. 2c) in the Greek population, an allergen that allowed identifying almost 90 % of children with generalized reactions to hazelnut [12]. This is in accordance with the fact that all patients sensitized to Cor a 14 were also sensitized to Cor a 9 [2]. Unfortunately, we didn’t assay corresponding IgE in individual sera.

Interestingly, the higher sensitization to Cor a 9 and Cor a 11 was observed in one of the 2 children of our population, i.e., patient #14, a 8-year old child from Athens. The other child, a 4-year-old from Madrid was monosensitized to Cor a 9 (patient #18). In our restricted population, none of the adults from the 2 birch-endemic regions were sensitized to these allergens. This is in accordance with the observation that sensitization to Cor a 11 was not or rarely found in hazelnut-allergic adults from birch-endemic region such as the Netherlands and Belgium [7, 22], whereas sensitization to Cor a 11 was evidenced in children of such area, mainly in infants demonstrating severe reaction. In birch-endemic region, Cor a 9 (and Cor a 14) sensitization was evidenced as soon as preschool age in infants with atopic dermatis, preceding sensitization to Cor a 11, and in children demonstrating severe symptoms [7, 11, 12].

The biologic activity of the 4 tested allergens was then analysed by histamine release assay, further confirming the sensitization patterns observed in EAST and IgE immunoblot, and the efficiency of the interaction between these sera and the corresponding specific allergen. We can observe that for some patients and allergens, the presence of specific IgE is not confirmed by HR. For example, rCor a 1.04 specific IgE were detected in 2 patients from Spain (patients #17 and #19), but were not associated with basophil activation. This can reflected IgE cross reactivity with others Bet v 1 pollen allergens, as these two patients were allergic to grass and olive or grass, cypress and plane tree pollens, but not clinical reactivity. The biological activity of nCor a 11 [7] or nCor a 9, rCor a 1 and rCor a 8 [6] was also studied using Basophil activation test and selected sera from birch-endemic region. In this study, the most active allergen, i.e., the allergen activating basophils at the lower dose, was Cor a 1. In the present study we demonstrated that the activity of rCor a 8 is very high in the Athens patients, with elicitation potency and BHR classes higher than that observed for the reference hazelnut extract. In contrast, HR release induced by rCor a 1.04 was far less efficient in patients from France and Switzerland. In these patients, the most efficient release was induced by the hazelnut extract, thus confirming the presence of other IgE reactive protein(s) for these patients in the extract. BHR classes for Cor a 9 and Cor a 11 in respective-sensitized patients were lower. Altogether, this thus suggests that rCor a 8 is a more potent elicitor than the other tested allergens, which can also partially explain the more severe symptoms in the patients sensitized to this allergen [4, 5].

## Conclusions

Although based on a limited number of patients, this work extends previously published researches on the sensitizing profiles of hazelnut allergic patients in birch-endemic versus Mediteranean regions and provides new data on the biological activity of hazelnut allergens and extracts. Notably, the present study demonstrates the high biological activity of Cor a 8 and the IgE reactivity and functionality of other pollen-related proteins in hazelnut extract.

## Additional file

Additional file 1: Figure S1. Basophil histamine release induced by hazelnut allergens using Strasbourg sera for sensitization. Stripped human basophils were passively sensitized with serum from patients from Strasbourg, France (Patients #2, #3 or #5) and then incubated with increasing concentrations of hazelnut allergens, i.e., hazelnut extract (black), rCor a 1 (red), rCor a 8 (blue), Cor a 9 (purple) or Cor a 11 (green). Released histamine is represented as percentage of reference release, i.e., induced by anti-IgE.
