# Supplementary figures and images for: Further studies on the biological activity of hazelnut allergens
Source: Clin Transl Allergy. 2015 Jul 17;5:26. doi: 10.1186/s13601-015-0066-7 (PMC4506444; doi:10.1186/s13601-015-0066-7)

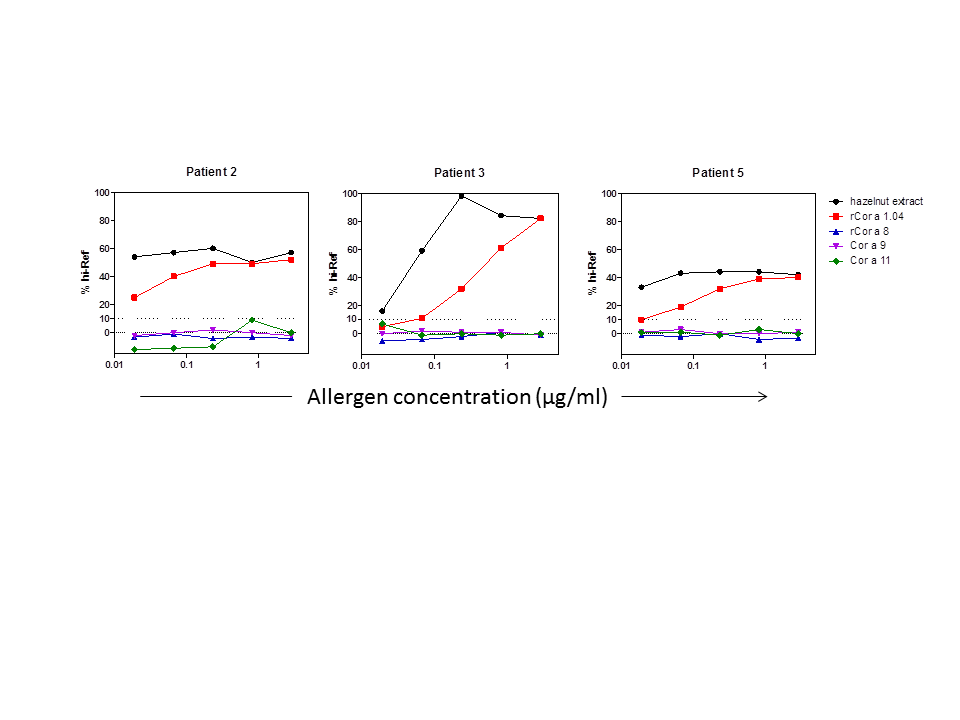

Supplement: Additional file 1: Figure S1. — Basophil histamine release induced by hazelnut allergens using Strasbourg sera for sensitization. Stripped human basophils were passively sensitized with serum from patients from Strasbourg, France (Patients #2, #3 or #5) and then incubated with increasing concentrations of hazelnut allergens, i.e., hazelnut extract (black), rCor a 1 (red), rCor a 8 (blue), Cor a 9 (purple) or Cor a 11 (green). Released histamine is represented as percentage of reference release, i.e., induced by anti-IgE. [file 13601_2015_66_MOESM1_ESM.tiff]
